# *Ki67*, *CD105*, and *α-SMA* Expression Supports Biological Distinctness of Oral Squamous Cell Carcinoma Arising in the Background of Oral Submucous Fibrosis

**DOI:** 10.31557/APJCP.2020.21.7.2067

**Published:** 2020-07

**Authors:** Amol Ramchandra Gadbail, Sheetal Korde, Minal S Chaudhary, Sachin C Sarode, Shailesh M Gondivkar, Ravi Dande, Satyajit Ashok Tekade, Monal Yuwanati, Alka Hande, Shankargouda Patil

**Affiliations:** 1 *Department of Dentistry, Indira Gandhi Government Medical College and Hospital, Nagpur, Maharashtra, India. *; 2 *Department of Oral Pathology and Microbiology, Yerala Dental College and Hospital, Kharghar, Mumbai, India. *; 3 *Department of Oral Pathology and Microbiology, Sharad Pawar Dental College and Hospital, Datta Meghe Institute of Medical Sciences, Sawangi (M), Wardha, Maharashtra, India. *; 4 *Department of Oral Pathology and Microbiology, Dr. D.Y. Patil Dental College and Hospital, Dr. D.Y. Patil Vidyapeeth, Sant-Tukaram Nagar, Pimpri, Pune. *; 5 *Department of Oral Medicine and Radiology, Government Dental College and Hospital, Nagpur, Maharashtra, India. *; 6 *Department of Oral Pathology and Microbiology, Modern Dental College and Research Centre, Gandhi Nagar, Indore, Madhya Pradesh, India. *; 7 *Department of Oral Pathology and Microbiology, People’s College of Dental Science & Research Centre, People’s University, Bhopal, Madhya Pradesh, India. *; 8 *Department of Maxillofacial Surgery and Diagnostic Sciences, Division of Oral Pathology, College of Dentistry, Jazan University, Jazan, Saudi Arabia. *

**Keywords:** Oral submucous fibrosis, oral squamous cell carcinoma, Ki67, CD105- α-SMA, areca nut

## Abstract

**Background:**

The clinicopathological distinctness of oral squamous cell carcinoma arising in the background of oral submucous fibrosis (OSCC-OSF) is well known; however, the molecular distinctness of this unique OSCC-OSF has not been investigated to date. With this in mind, we compared the expression of *Ki67*, *CD105*, and *α-SMA* between OSCC-OSF and oral squamous cell carcinoma (OSCC).

**Methods:**

Formalin-fixed paraffin-embedded tissues of 105 OSCC-OSF and 112 OSCC cases were subjected to immunohistochemistry for evaluation of *Ki67*, *CD105*, and *α-SMA* expression.

**Results:**

*Ki67* (labeling index) *LI*, *MVD* and *α-SMA* expression were significantly higher in OSCC compared to OSCC-OSF.* Ki67 LI* and *MVD* was significantly higher in OSCC compared to OSCC-OSF in parameters such as well-differentiated, early TNM stage, non-metastatic, and more than 3-year survival. α-SMA expression was significantly higher in OSCC compared to OSCC-OSF in parameters such as moderate differentiation, metastatic lesions, and survival less than 3 years. *Ki67 LI*, *MVD* and *α-SMA* showed significant positive correlation with each other in OSCC and OSCC-OSF.

**Conclusion:**

Proliferation, neoangiogenesis and myofibroblast differentiation were significantly higher in the OSCC group compared to the OSCC-OSF group. This suggests the biological distinctness of OSCC-OSF, which could help the future development of targeted therapies.

## Introduction

In the Indian subcontinent, prevalent use of areca nut alone or in combination with tobacco, lime, slaked lime and betel leaves was predominantly associated with oral submucous fibrosis (OSF). OSF has been defined by the World Health Organization (WHO) as a “slowly progressive disease, in which the fibrous bands form in the oral mucosa, ultimately leading to severe restriction of movement of the mouth, including the tongue” (WHO, 1984). In a recent study, the malignant transformation rate for OSF was 3.72% (Wang et al., 2004).Thus, oral squamous cell carcinoma (OSCC) arising in the background of OSF (OSCC-OSF) is one of the most common malignancies predominantly in India and parts of South and Southeast Asian countries (Chiba, 2001).

OSCC-OSF has certain peculiar clinicopathological characteristics, which include younger age group, male predilection, better tumor differentiation grade, less cervical lymph node metastasis, extracapsular spread, early detection, and better prognosis (Chaturvedi et al., 2013; Sarode and Sarode, 2013; Gadbail et al., 2017).Moreover, there is a less frequent requirement of adjuvant therapy compared to conventional OSCC (Chaturvedi et al., 2017). Although, there is reported clinicopathological distinctness, the molecular distinctness of OSCC-OSF has not been investigated to date. Apart from aforementioned clinico-pathological heterogeneity in OSCC-OSF, there is strong possibility of existence of heterogeneity at molecular level. Understanding molecular diversification is important for future development of targeted therapy. Apart from 5-year survival rate estimation, such distinction will also help in establishing prognostic markers for predicting loco-regional as well as distant metastasis.

With this view in mind, a comparative study was designed to investigate the immune-expression of *Ki67*, *CD105* and *α-SMA* in OSCC-OSF and OSCC with clinicopathological correlation.

## Materials and Methods

The present study was carried out at the Department of Oral and Maxillofacial Pathology and Microbiology at Sharad Pawar Dental College and Hospital, Wardha, Maharashtra, India. The ethical approval for this studywas obtained from Institutional ethics committee of Datta Meghe Institute of Medical Sciences, Wardha, India (Ref no. DMIMS (DU)/IEC/2014-15/953, dated 15/12/2014). Archival samples of study group and the relevant data were retrieved from the archival of the department. Patients with clinical and histopathological evidence of OSCC and undergone surgical excision with or without radiation were included in the present study. The OSCC-OSF cases were categorized based on the following clinical criteria: intolerance to hot and spicy foods, pale looking oral mucosa, palpable fibrotic bands, and chronic progressive trismus. Additionally, histopathological evidence of OSF was also considered for confirmation of OSCC-OSF group. The patients were staged according to the clinical TNM staging system as mentioned by American Joint Committee of Cancer staging manual 2002 (Greene et al., 2002). Histopatholigical examination was used for confirmation of nodal metastasis. Patients with a history of previous OSCC, recurrent or distant disease and pre-operative chemotherapy, radiotherapy, or surgery (other than a biopsy) were excluded from the study. All the included cases were then followed up prospectively for obtaining disease free survival data of more than 3 years. The details of immunostochemistry of OSCC-OSF cases included in this study were taken from our previous published paper (Gadbail et al, 2019).

*Immunohistochemistry*

The standard specific antigen retrieval was carried out for *Ki67* and *α-SMA* using 0.01 M sodium citrate buffer (pH 6.0), except for *CD105* using Proteinase K for 5 min. Blocking of endogenous peroxidase activity was done with 3% H_2_O_2_ in methanol for 30 min. Prevention of non-specific reaction was performed by incubating slides with 10% serum for 10 minutes. Pre-diluted α-SMA antibody [Monoclonal Mouse Anti-Human (MMAH), Clone:1A4; Product code (PC):IR611, Dako, Denmark (DD)], pre-diluted *Ki67* antibody (clone MIB-1; PC:N1633; DD), and CD105 antibody (Diluted 1:30, MMAH, Clone:SN6h,PC:M3527, DD) were incubated at room temperature in a humidifying chamber for 60 min. Appropriate positive controls were used for α-SMA (myoepithelial cells of the salivary gland), CD105 (pyogenic granuloma) and *Ki67* (hyperplastic lymphnode). The HRP-labeled Polymer Anti-mouse secondary antibody (DakoEnVision System, PC: K4000, DD.) was incubated at room temperature in a humidifying chamber for 30 min. The freshly prepared substrate/chromogen solution of 3, 3’ Diaminobenzidine in the provided buffer was used to visualize the antigen-antibody reaction and counterstained in Mayer’s hematoxylin.

*Immunohistochemistry scoring*

Evaluations of expression of all the markers were performed at the invasive tumor front areas of the sections.

*Assessment of Ki67 positive cells*

Neoplastic cells showing brown stained nuclei were considered as positive cells. The most heavily Ki67 labeled area was located by scanning the sections at 100 X magnification. Cell counts were made at 400X magnification in 5 randomly selected fields. The number of positively stained nuclei was expressed as a percentage of the total number counted epithelial cells. *Ki67* labeling index (LI) = number of IHC positive cells × 100/ total number of cells observed (Gadbail et al., 2017). 

*Assessment of CD105 positive cells*

CD105 positive vascular endothelial cells were identified by their brown cytoplasmic staining. Areas with evidence of inflammation were avoided for assessment of CD105 positive cells. Criteria given by Weidner et al.,1991 was used for counting the microvessels. A highlighted endothelial cell or a cell cluster clearly separated from adjacent microvessels and other connective tissue elements was regarded as a distinct countable microvessel. Moreover, single cell sprouts were included in the count. After scanning, the mean vascular density (MVD) was measured by counting CD-105 positive vessels in two hotspot areas at 100X using Leica Qwin standard software and Leica DM LB 2 research microscope. The MVD was assessed in invasive tumor front area.

*Assessment of α-SMA positive cells*

Irrespective of the intensity of staining, intra-cytoplasmic stained cells (other than non-inflammatory and non-endothelial) with α-SMA were regarded as myofibroblasts. Methodology applied by Etemad Moghadam et al.,(2009) was used for evaluation α-SMA score. The percentage of cells positive for α-SMA in the tumor stroma was recorded as: 0 = no positive cells, 1 = 1–33% positive cells, 2 = 34–66% positive cells, and 3 = 67–100% positive cells by three observers. The same score obtained by more than two observers was counted as the final score. Scores 1, 2, and 3 were graded as mild, moderate, and intense expression of α-SMA, respectively. 

*Statistical analysis*

The data were statistically analyzed using SPSS, version 17.0 for Windows. Student’s t-test and Mann-Whitney U tests were applied for the differences in *Ki67 LI*, *MVD *and *α-SMA* between the OSCC and OSCC-OSF in clinicopathological parameters. The level of statistical significance was at p<0.05. The correlation was carried out among *Ki67 LI, MVD* and* α-SMA* expression by using the Pearson’s rank correlation analysis test with the level of statistical significance at p<0.01.

## Results

*Demographic data*

The study included 217 patients of which 112 (51.61%) had conventional OSCC without OSF (OSCC) and 105 (48.38%) had OSCC with OSF (OSCC-OSF). In the OSCC-OSF group, 45.71% and 54.29% of the cases were presented in early (stage I and II) and advanced (Stage III and IV) clinical TNM stage, respectively. In contrast, in the OSCC group, 17.85% and 82.15% of the cases were presented in the early stage and advanced stage, respectively. In the OSCC-OSF group, the majority of the cases were well-differentiated (WD) (60.95%) followed by moderately differentiated (MD) (34.28%) and poorly differentiated (PD) (4.76%). In the OSCC group, the majority of cases were MD (53.57%) followed by WD (38.39%) and PD (8.03%). Regional lymph node metastasis was observed in 27.61% and 41.07% cases of OSCC-OSF and OSCC group, respectively. The three-year disease free survival rate was 78.09%and 58.92% cases of OSCC-OSF and OSCC group, respectively.

3.2 Immunohistochemistry

Statistically, *Ki67 LI*, *MVD* and *α-SMA* were significantly higher in OSCC compared to OSCC-OSF (p<0.001) (Table 1). The *α-SMA* expression was not observed in 8 (7.14%) of OSCC and 14 (13.33%) of OSCC-OSF. *Ki67 LI* and *MVD*; *Ki67 LI* and* α-SMA*; and *α-SMA* and *MVD* showed statistically significant positive correlations in OSCC and OSCC-OSF.

**Figure 1:**
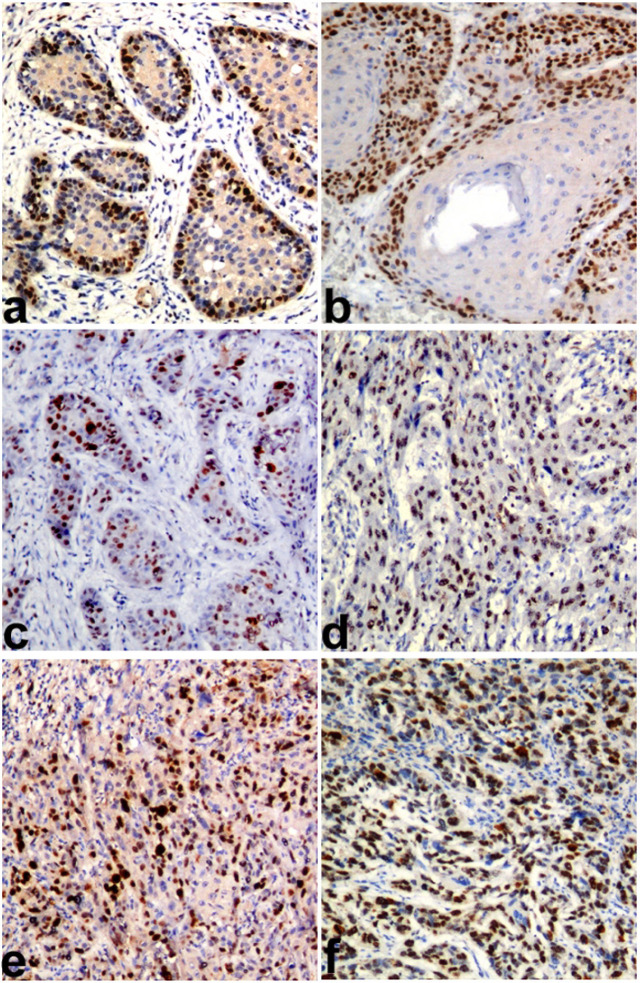
Photomicrograph Showing *Ki67* Antigen Expression (*KI67 LI*) in a) WDSCC of OSCC-OSF, b) WDSCC of OSCC, c) MDSCC of OSCC-OSF, d) MDSCC of OSCC, e) PDSCC of OSCC-OSF, and f) PDSCC of OSCC. (Immunohistochemistry; Magnification X100).

**Figure 2:**
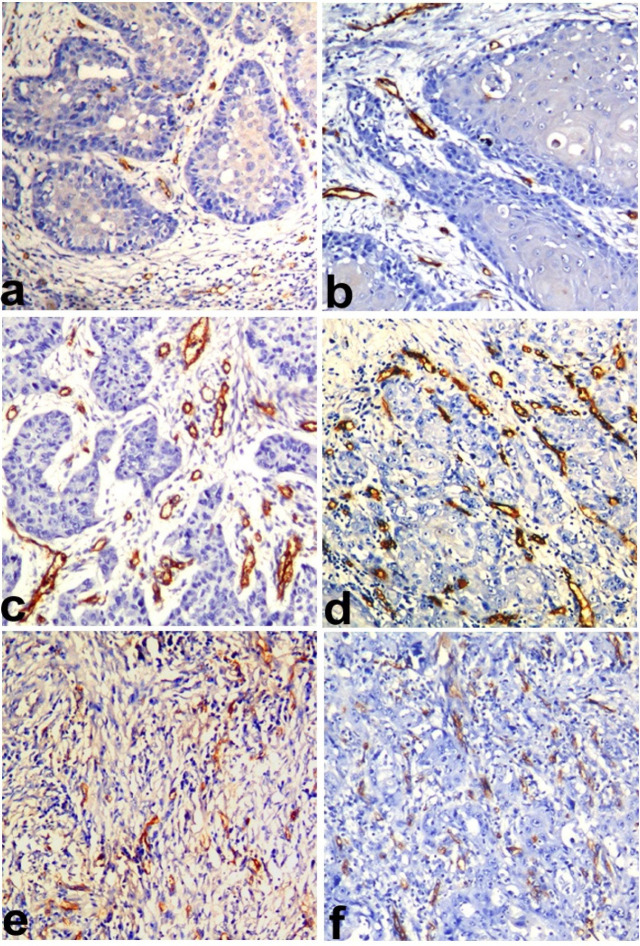
Photomicrograph Showing CD105 Antigen Expression (MVD) in a) WDSCC of OSCC-OSF, b)WDSCC of OSCC, c) MDSCC of OSCC-OSF, d) MDSCC of OSCC, e) PDSCC of OSCC-OSF, and f) PDSCC of OSCC. (Immunohistochemistry; Magnification X100

**Figure 3:**
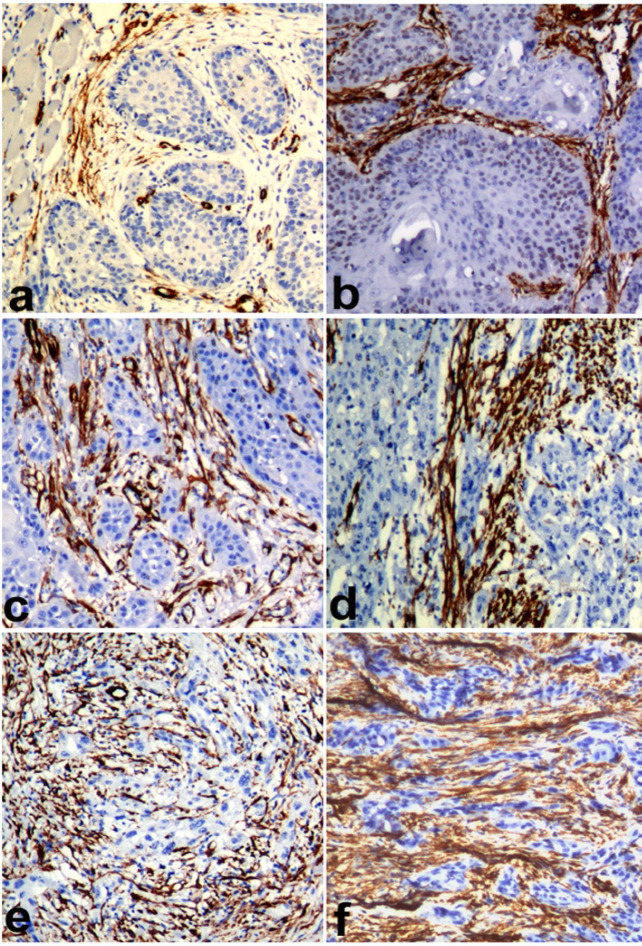
Photomicrograph Showing α-SMA Expression in a) WDSCC of OSCC-OSF, b)WDSCC of OSCC, c) MDSCC of OSCC-OSF, d) MDSCC of OSCC, e) PDSCC of OSCC-OSF, and f) PDSCC of OSCC. (Immunohistochemistry; Magnification X100)

*Ki67 LI* was statistically significantly higher in WD OSCC compared to WD OSCC-OSF (p=0.018). Similarly, the MD and PD OSCC showed more *KI67 LI *compared to the MD and PD OSCC-OSF, but the results were not statistically significant (Figure 1). MVD was observed to be statistically significantly higher in WD OSCC compared to WD OSCC-OSF (p<0.001). Statistically non-significant differences in MVD were noted in MD (p=0.905) and PD (p=0.714) squamous cell carcinoma between OSCC and OSCC-OSF (Figure 2). α-SMA expression was observed to be statistically significantly higher in MD OSCC compared to MD OSCC-OSF (p=0.046). Statistically non-significant differences in the α-SMA expression were noted in WD (p=0.457) and PD (p=1.00) squamous cell carcinoma between OSCC and OSCC-OSF (Figure 3) (Table 2).

*Ki67 LI* were observed to be statistically significantly higher in early clinical TNM stage of OSCC compared to OSCC-OSF (p=0.015). Similarly, advanced clinical TNM stages in the OSCC group showed a higher *Ki67* *LI* compared to the OSCC-OSF group, but the differences were statistically insignificant (p=0.153). In advanced and early stages, both the MVD and α-SMA expression were higher in the OSCC group compared to the OSCC-OSF group, but the differences were statistically insignificant (Table 3).

*Ki67 LI *was observed to be statistically significantly higher in non-metastatic OSCC compared to OSCC-OSF (p=0.007). Similarly, *Ki67 LI* was observed to be statistically significantly higher in metastatic OSCC compared to OSCC-OSF (p=0.038). In non-metastatic cases, MVD was noted to be statistically significantly higher in OSCC compared to OSCC-OSF (p < 0.001). Although MVD was higher in metastatic OSCC than metastatic OSCC-OSF, the differences were not statistically significant. In metastatic cases, α-SMA expression was noted to be statistically significantly higher in the OSCC group compared to OSCC-OSF (p=0.011). Similarly, in non-metastatic cases, α-SMA expression was noted to be higher in the OSCC group compared to the OSCC-OSF but the differences were not statistically significant (Table 3).

In patients with more than a 3-year survival, *Ki67 LI *was significantly higher in the OSCC group compared to the OSCC-OSF group (p=0.013). Similarly, MVD noted a statistically significantly higher in-patient survival of more than 3 years of OSCC compared to OSCC-OSF (p=0.001). *Ki67 LI *and *MVD* showed insignificant differences in patients who did not survive more than 3 years of OSCC and OSCC-OSF. α-SMA expression was noted to be significantly higher in patients that did not survive more than 3 years of OSCC compared to OSCC-OSF (p=0.036). However, a statistically non-significant difference of α-SMA expression was noted in patients who survived more than 3 years of OSCC and OSCC-OSF. (Table 3)

## Discussion

The role of the areca nut as a carcinogen has been proven beyond a doubt, with many animal studies demonstrating its carcinogenicity, mutagenicity, and genotoxicity. Studies on the molecules implicated in cell cycle regulation, hypoxia, processes leading to DNA double-strand breaks, senescence, and many other pathways related to carcinogenesis have shown ample evidence for malignant transformation in OSF induced by the areca nut (Ekanayaka and Tilakaratne, 2016). More importantly, OSCC-OSF is now considered a clinicopathologically distinct disease, and the differences are believed to arise from the differential mechanisms of areca nut carcinogenesis (Chaturvedi et al., 2013; Sarode and Sarode, 2013; Chaturvedi et al., 2017; Gadbail et al., 2017). Thus, there is a dire need for the investigation of the molecular expression in OSCC-OSF to better understand the pathogenesis. In the resent study, we evaluated the intrinsic growth potential using a *Ki67* antibody (proliferation marker) (Brown and Gatter, 2002), tumor neoangiogenesis (*CD105*) (Dallas et al., 2008)and myofibroblast (α-SMA) expression (myofibroblast important role in invasion and metastasis) (Giatromanolaki et al., 2007) in OSCC and OSCC-OSF with respect to clinicopathological parameters.

In the present study, *Ki67 LI* expression was significantly higher in OSCC compared to OSCC-OSF. Furthermore, in groups such as WD, early TNM stage, non-metastatic, metastatic, and more than 3-year survival of *Ki67 LI* was significantly higher in the OSCC compared to OSCC-OSF. As, *Ki67* is cell proliferation and prognostic marker of OSCC (Da Ros Motta et al., 2009; Wang et al., 2009),an OSCC-OSF appeared to be less aggressive compared to OSCC and thus complimented the clinicopathological distinctness reported in previous literature (Chaturvedi et al., 2013; Chaturvedi et al., 2017; Gadbail et al., 2017). These results could be attributed to the areca nut-specific molecular pathway activation during tumorigenesis. In the OSCC group, *Ki67 LI* was higher in the MD and PD parameters, advanced TNM stage, metastasis (cervical lymph node) and less than 3-year survival. However, there were no statistically significant differences between the OSCC and OSCC-OSF group in these parameters. It is difficult to draw any conclusion for these findings and further studies at the proteomic and genomic levels are recommended to further authenticate the proliferation related distinctness between the two groups.

The activation of angiogenesis plays a key role in the initiation of growth and metastatic process in cancers (Weidner et al., 1991). Thus, the assessment of the microvessel density (MVD) is a reliable marker of the assessment of tumor biological aggressiveness and its clinical prognostic outcome (Pruneri et al., 2002). *CD105 *is a hypoxia-induced protein and specifically reacts with proliferating endothelial cells in tissues undergoing active angiogenesis, which signifies tumorigenic neo-angiogenesis (Dallas et al., 2008). *CD105* expression in OSCC is a promising parameter for assessing the patients at greater risk of recurrent malignancy, lymph node metastasis, and prognosis could bear anti-angiogenic therapeutic potential (Marioni et al., 2010). In the present study, MVD was observed to be statistically significantly higher in OSCC compared to OSCC-OSF. Furthermore, MVD was significantly higher in parameters such as WD, early TNM stage, non-metastatic and more than 3-year survival. Comparatively less *MVD* expression in OSCC-OSF could be attributed to fibrosis in the tumor stroma that can cause the reduction/blocked of vascularity (Tilakaratne et al., 2006). It is well established that tumor progression in OSCC depends on the co-occurrence of angiogenesis and cell proliferation (Ravi et al., 1998; Macluskey et al., 2000). In agreement with this, we reported a positive correlation between *MVD* and *Ki67LI *in both the groups. The results of the present study cannot rule out the role of the autocrine or paracrine activity of angiogenesis related growth factors for reduced vascularity in the OSCC-OSF group. The above results also depict the distinctness of OSCC-OSF groups in terms of tumor associated neoangiogenesis. In contrast, tumor neoangiogenesis showed no significant differences between OSCC-OSF and OSCC with respect to PD, advanced TNM stage, metastasis, and <3-year survival. 

Cancer associated myofibroblasts are one of the most important mesenchymal cells in the tumor microenvironment, which play multiple roles in promoting tumor growth, invasion and metastasis (Cirri and Chiarugi, 2011). The myofibroblast proliferation in the microenvironment plays a crucial role in the epithelial-mesenchymal transition in OSCC, thus causing tumor invasion, the occurrence of occult neck disease, distant metastasis and poor survival in oral cancer (Luksic et al., 2015; Lin et al., 2017). In the present study, *α-SMA *expression was significantly higher in the OSCC group compared to the OSCC-OSF group. Likewise,* α-SMA* expression was significantly higher in OSCC compared to OSCC-OSF in parameters such as MD, metastatic lesions, and survival less than 3 years. This result indicates that the OSCC-OSF shows minimal carcinogenesis relevant changes in the tissue microenvironment compared to OSCC. Moreover, it also advocates the molecular distinctness of OSCC-OSF in terms of myofibroblast expression and related signaling events. In the present study, non-significant differences of *α-SMA* expression was noted between OSCC and OSCC-OSF with respect to WD, clinical TNM staging, and non-metastatic patients that survived more than 3 years. However, *α-SMA *expression in the aforementioned parameters was higher in OSCC compared to OSCC-OSF. We recommend future studies on myofibroblast associated signaling pathways to further explore its role in the tumorigenesis of OSCC-OSF.

The IHC expressions of the protein do not necessarily suggest the active status of the protein. This is inherent demerit of the IHC technique and hence, it should be supported by other molecular investigations at genomic and proteomic level. In the present study, we compared the expression of three markers (*Ki67*,* CD105*, and *α-SMA*) between OSCC-OSF and OSCC. Holistic point of view, there is vast array of cross talk between these markers other carcinogenetically relevant molecules. Hence, in depth analysis of each molecule with regards to particular hallmark of carcinogenesis is warranted in future research. Future studies are also recommended on designing predictive biomarker kit based on the finding of the present study that will guide in treatment planning and determination of prognosis. 

Literature search depicts that OSCC-OSF shows better prognosis as compared to OSCC alone. This is mainly attributed to better grade of tumor differentiation, less LN metastasis and better 3 year survival in OSCC-OSF patients (Chaturvedi et al., 2013; Sarode and Sarode, 2013; Gadbail et al., 2017). These clinical picture points out towards less aggressive biological behavior of the OSCC-OSF tumor, which also means less expression of carcinogenesis relevant biological markers. In tuned with this, we observed that proliferation, neoangiogenesis and myofibroblast differentiation were significantly higher in the OSCC group compared to the OSCC-OSF group.

In conclusion, this study is the first of its kind to attempt to investigate the biological distinctness of OSCC-OSF. This unique malignancy showed significantly less expression of *Ki67* *LI*,* CD105* and *α-SMA* compared to conventional OSCC. Since, these three markers are interlinked, a positive correlation between the immune expression of *Ki67 LI*, *MVD* and *α-SMA *in OSCC, as well as OSCC-OSF was observed. Due to lower expression of the aforementioned markers, OSCC-OSF could be a biologically less aggressive entity compared to non-OSF associated OSCC. We believe that upstream and downstream signaling events related to these markers could also be altered in the OSCC-OSF group, which warrants in depth analysis in the future. One can attribute this to the unique carcinogenicity of the areca nut and its constituents. A future study in this regard will help us to better understand the molecular events involved in the pathogenesis of OSCC-OSF. Moreover, this would also prove the biological distinctness of OSCC-OSF, which will help in the development of future targeted therapies.

**Table 1:** Comparison of *Ki67, CD105 (MVD)* and *α-SMA *Expression between OSCC and OSCC-OSF

Bio-markers	Groups	n	Mean (SD)	P-value
*Ki67 LI*	OSCC	112	55.84 (12.22)	<0.001
	OSCC-OSF	105	49.87 (11.25)	
*MVD*	OSCC	112	82.44 (14.34)	<0.001
	OSCC-OSF	105	74.32 (14.62)	
*α-SMA*	OSCC	112	2.05 (0.94)	<0.001
	OSCC-OSF	105	1.70 (0.88)	

n, number; SD, Standard deviation; *Ki67 LI*,* Ki67* Labeling Index; *MVD*, Mean Vascular Density; OSCC, Oral squamous cell carcinoma; OSCC-OSF, Oral squamous cell carcinoma arising in the background of Oral submucous fibrosis; *α-SMA*, Alpha smooth muscle actine.

**Table 2:** Comparison of *Ki67*, *CD105 (MVD)* and *α-SMA* Expression between OSCC, and OSCC-OSF with Respect to Histopathologicalgrading (HPG)

HPG	Biomarkers	groups	n	Mean (SD)	*P*-value
WDSCC	*Ki67*	OSCC	37	45.07 (4.66)	0.018
		OSCC-OSF	63	42.93 (5.50)	
	*MVD*	OSCC	37	73.94 (10.90)	<0.001
		OSCC-OSF	63	65.63 (8.20)	
	*α-SMA*	OSCC	37	1.21 (0.85)	0.457
		OSCC-OSF	63	1.33 (0.80)	
MDSCC	*Ki67*	OSCC	69	59.79 (10.55)	0.48
		OSCC-OSF	37	58.71 (8.14)	
	*MVD*	OSCC	69	85.36 (13.51)	0.905
		OSCC-OSF	37	85.78 (11.47)	
	*α-SMA*	OSCC	69	2.42 (0.69)	0.046
		OSCC-OSF	37	2.16 (0.68)	
PDSCC	*Ki67*	OSCC	6	76.85 (6.48)	0.361
		OSCC-OSF	5	71.87 (11.91)	
	*MVD*	OSCC	6	101.33 (12.24)	0.714
		OSCC-OSF	5	99.00 (13.07)	
	*α-SMA*	OSCC	6	3.00 (0.00)	1.00
		OSCC-OSF	5	3.00 (0.00)	

Histopathological grading, HPG; WDSCC, Well differentiated squamous cell carcinoma; MDSCC, Moderately differentiated squamous cell carcinoma; PDSCC, Poorly differentiated squamous cell carcinoma

**Table 3:** Comparison of *Ki67, CD105 (MVD)* and *α-SMA* Expression between OSCC, and OSCC-OSF with Respect to Clinical Features (Clinical TNM Stages, Metastasis and 3 Years Survival)

Clinical Parameters	Biomarkers	groups	n	Mean (SD)	*P*-value
Early stage (Clinical TNM stage)	*Ki67*	OSCC	20	53.90 (14.47)	0.015
		OSCC-OSF	48	45.56 (10.31)	
	*MVD*	OSCC	20	74.85 (14.33)	0.059
		OSCC-OSF	48	67.62 (12.01)	
	*α-SMA*	OSCC	20	1.45 (1.05)	0.588
		OSCC-OSF	48	1.29 (0.84)	
Advanced Stage (Clinical TNM stage)	*Ki67*	OSCC	92	56.26 (11.72)	0.153
		OSCC-OSF	57	53.49 (10.79)	
	*MVD*	OSCC	92	84.09 (13.88)	0.05
		OSCC-OSF	57	79.96 (14.33)	
	*α-SMA*	OSCC	92	2.18 (0.87)	0.207
		OSCC-OSF	57	2.05 (0.76)	
Non metastatic	*Ki67*	OSCC	65	51.27 (11.09)	0.007
		OSCC-OSF	76	47.10 (10.28)	
	*MVD*	OSCC	65	77.40 (13.28)	<0.001
		OSCC-OSF	76	69.52 (11.38)	
	*α-SMA*	OSCC	65	1.64 (0.97)	0.39
		OSCC-OSF	76	1.51 (0.87)	
Metastatic	*Ki67*	OSCC	47	62.16 (10.90)	0.038
		OSCC-OSF	29	57.11 (10.58)	
	*MVD*	OSCC	47	89.42 (12.86)	0.335
		OSCC-OSF	29	86.89 (14.87)	
	*α-SMA*	OSCC	47	2.61 (0.53)	0.011
		OSCC-OSF	29	2.20 (0.72)	
Survived more than 3years	*Ki67*	OSCC	66	51.50 (11.36)	0.013
		OSCC-OSF	82	47.49 (10.38)	
	*MVD*	OSCC	66	77.69 (13.94)	0.001
		OSCC-OSF	82	70.10 (11.75)	
	*α-SMA*	OSCC	66	1.62 (0.95)	0.507
		OSCC-OSF	82	1.52 (0.86)	
Survived less than 3 years	*Ki67*	OSCC	46	62.06 (10.71)	0.183
		OSCC-OSF	23	58.34 (10.28)	
	*MVD*	OSCC	46	89.26 (12.09)	0.794
		OSCC-OSF	23	89.34 (14.17)	
	*α-SMA*	OSCC	46	2.67 (0.47)	0.036
		OSCC-OSF	23	2.34 (0.64)	

## Declaration

The study protocol was approved by the institutional ethics committee of DattaMeghe Institute of Medical Sciences, Wardha, India (Ref no. DMIMS (DU)/IEC/2014-15/953, dated 15/12/2014).
